# Altered cardiac autonomic nervous function in depression

**DOI:** 10.1186/1471-244X-13-187

**Published:** 2013-07-10

**Authors:** Yiming Wang, Xun Zhao, Adrienne O'Neil, Alyna Turner, Xingde Liu, Michael Berk

**Affiliations:** 1 Department of Psychiatry, The Affiliated Hospital of Guiyang Medical University, Guiyang, Guizhou 550004, China; 2 IMPACT Strategic Research Centre, School of Medicine, Deakin University, Geelong 3220, Australia; 3 Affiliated Hospital of Jining Medical University, Jining, Shangdong 250000, China; 4 School of Public Health and Preventive Medicine, Monash University, Melbourne 3004, Australia; 5 Department of Cardiology, The Affiliated Hospital of Guiyang Medical University, Guiyang, Guizhou 550004, China; 6 Department of Psychiatry, Centre of Youth Mental Health, The University of Melbourne, Parkville, Melbourne, VIC 3052, Australia; 7 Florey Institute of Neuroscience and Mental Health, University of Melbourne, Parkville, Melbourne, VIC 3010, Australia

**Keywords:** Depression, Cardiac autonomic nervous system, Heart rate variability, Arrhythmia, Vagus, Cardiovascular disorders

## Abstract

**Background:**

Depression is an independent risk factor for coronary artery disease. Autonomic instability may play a mediating or moderating role in this relationship; however this is not well understood. The objective of this study was to explore cardiac autonomic function and cardiac arrhythmia in depression, the correlation between depression severity and Heart Rate Variability (HRV) related indices, and the prevalence of arrhythmia.

**Methods:**

Individuals (n = 53) with major depression as assessed by the Diagnostic and Statistical Manual of Mental Disorders, who had a Hamilton Rating Scale for Depression (HAMD) score ≥20 and a Zung Self-Rating Depression Scale score > 53 were compared to 53 healthy individuals, matched for age and gender. Multichannel Electrocardiograph ECG-92C data were collected over 24 hours. Long-term changes in HRV were used to assess the following vagally mediated changes in autonomic tone, expressed as time domain indices: Standard deviation of the NN intervals (SDNN), standard deviation of 5 min averaged NN intervals (SDANN), Root Mean Square of the Successive Differences (RMSSD) and percentage of NN intervals > 50 ms different from preceding interval (pNN50). Pearson’s correlations were conducted to explore the strength of the association between depression severity (using the SDS and HRV related indices, specifically SDNN and low frequency domain / high frequency domain (LF/HF)).

**Results:**

The values of SDNN, SDANN, RMSSD, PNN50 and HF were lower in the depression group compared to the control group (*P*<.05). The mean value of the LF in the depression group was higher than the in control group (*P*<.05). Furthermore the ratio of LF/HF was higher among the depression group than the control group (*P*<.05). A linear relationship was shown to exist between the severity of the depression and HRV indices. In the depression group, the prevalence of arrhythmia was significantly higher than in the control group (*P*<.05), particularly supraventricular arrhythmias.

**Conclusions:**

Our findings suggest that depression is accompanied by dysfunction of the cardiac autonomic nervous system, and further, that depression severity is linked to severity of this dysfunction. Individuals with depression appear to be susceptible to premature atrial and/or ventricular disease.

## Background

Prospective research has shown a significant relationship between depression and cardiovascular disease (CVD) [1]. In patients with coronary artery disease (CAD), co-occuring depression is associated with a range of poorer outcome measures [2-4] and a 2–4 fold increase in cardiac mortality and morbidity [5-9]. Depression has also recently emerged as a risk factor for the onset of CVD. Individuals with a history of depression are four times more likely to have a heart attack (myocardial infarction; MI) than those without [10]. However, the underlying physiology of this relationship remains poorly understood.

Data from animal models (e.g. the chronic mild stress (CMS) model) have indicated that depression exascerbates stress reactivity, and is associated with autonomic and cardiovascular dysfunction, including reduced heart rate variability (HRV) as well as elevated heart rate (HR) and sympathetic cardiac tone [11-13]. Studies conducted in human populations have also reported a relationship between cardiac autonomic dysfunction and altered cardiovascular reactivity [14]. While the pathophysiologic mechanisms linking cardiovascular events to depression are unclear, there is evidence implicating lower HRV, sympathetic tone changes, arrhythmias, and changes in ventricular electrophysiological properties [15]. HRV in particular is of increasing interest, due to its clinical application [16], and has been used as an index of cardiac autonomic functioning in patients with psychiatric disorders, personality or communication disorders.

To date, studies examining HRV in patients with existing CVD have produced inconsistent results. Several studies have found that reduced HRV in patients with post-MI depression [17,18] is linked to increased cardiac mortality, whereas other studies have shown no relationship between depression and low HRV in patients with stable CAD [16]. Exacerbating these inconsistencies is evidence that anxiety, which commonly co-occurs with depression, is associated with changes to HRV, and is implicated as an independent predictor of reduced parasympathetic activity in MI patients [19].

In order to elucidate the association between depression and cardiac autonomic nervous function, a comprehensive investigation of autonomic nervous system dysfunction in psychiatric populations is required. Patients with depression often suffer from symptoms of autonomic nervous system dysfunction such as palpitations occurring with a normal sinus rhythm as measured by electrocardiogram (ECG). Importantly, autonomic dysfunction and abnormal microvolt T wave alternans in these patients can increase the probability of sudden death [20]. We therefore sought to investigate cardiac autonomic function among individuals with and without depression, to determine the correlation between depression severity and HRV-related indices and to assess the prevalence of arrhythmia in this population.

## Methods

### Subjects

The depression group consisted of 53 patients (25 male, 28 female; age range 20–65, mean 40.9 ± 10.5), who were hospitalized for major depression in the Psychiatry Department of the affiliated hospital of Guiyang Medical University between March and December, 2012.

Study participants were those with a primary diagnosis of depression. To be eligible for inclusion in the study, all subjects met diagnostic criteria for depression as assessed by the Structured Clinical Interview for Diagnostic and Statistical Manual of the American Psychiatric Association-IV-TR (DSM-IV-TR) Axis I disorders (SCID). In addition, they were assessed using the Hamilton Rating Scale for Depression (HAMD), the Zung Self-rating Depression Scale (SDS) and Hamilton Anxiety Scale (HAMA). Inclusion criteria were a HAMD ≥ 20, a SDS > 53 and a depression severity index range of 0.25-1.0. Exclusion criteria included a HAMA score >14 points (to exclude patients with primary anxiety disorders); a diagnosis of an organic depressive disorder or substance dependence; or a history of any other psychiatric illness. Patients with a current or past history of acute myocardial infarction (as assessed by ECG testing) or comorbid medical conditions including diabetes mellitus were also excluded. Anti-depressant medication usage was not an exclusion criterion, as all patients were receiving SSRIs treatment.

The control group (n = 53, M 27, F 26; age range 20–63, mean 42.1 ± 11.61) consisted of normal healthy volunteers with a HAMD score <20, a HAMA score <7, and no history of depression, anxiety or somatic disorders.

### Consent

All participants gave their written informed consent to participate in the study. The procedures followed were in accordance with the revised edition of the Declaration of Helsinki of 1964 [21].

### Ethics

The study was approved by the ethics Committee on the Guidelines for Experiment at Guiyang Medical University (NO 04/2012).

### Assessment

#### Experiment processes

All participants were familiarized with the research protocol. Participants were precluded from taking any agents capable of influencing heart rate 48 hours prior to the experiments, such as Beta Blockers, cigarettes, alcohol, tea and coffee drinks, or from undertaking strenuous exercise within 24 hours of the assessments. Prior to the procedures, participants were asked to stay in a quiet room and rest for 15 minutes. Electrodes were attached and results analyzed by the same doctor.

#### Dynamic electrocardiogram collection

Twenty four hour ambulatory ECG data for all subjects was collected between 8:00 am to 8:00 am the next morning. Assessment of arrythmias was conducted by a technician who was blind to group allocation. HRV parameter values were analyzed using the ECG-92C multichannel electrocardiograph (Shanghai Photoelectric Electronic Medical Instruments Co., Ltd.). Paroxysmal tachycardia was diagnosed using repeated ECG readings (3+).

#### The depression severity index

The Zung Self-rating Depression Scale (SDS) is a 20-item self-report questionnaire that was used to assess depression severity. The total raw score is calculated by summing all items. The SDS depression severity index is calculated by the formula “SDS Index = Raw Score/80” [22].

#### Time and frequency domain index absolute measures of HRV

HRV, the physiological phenomenon where the sinus cardiac rhythm varies from the mean heart rate, is measured over a 24 hour monitoring period. It is a quantitative, non-invasive and highly reproducible index of cardiac autonomic function and an important indicator of the balance of sympathetic and parasympathetic tone. It reflects the autonomic nervous control of heart function by a dynamic controlling effect [23]. Time-domain analysis indices include SDNN, SDANN, RMSSD and PNN50 by the beat-to-beat or NN intervals. The SDNN (Standard deviation of the NN intervals) is an overall measure of HRV size and reflects all long-term components, indicative of dysregulation of the parasympathetic and sympathetic nerve function. SDANN (standard deviation of 5 min averaged NN intervals) is a sensitive index of the variability of the average of 5 minute periods, and reflects circadian influences including changes in position and physical activity. Both RMSSD (Root Mean Square of the Successive Differences) and pNN50 (percentage of NN intervals > 50 ms different from preceding interval) are linked to short-term changes in HRV rather than diurnal variations, and reflect vagally mediated changes in autonomic tone.

Frequency domain (power spectral density) analysis indices [24,25] at different amplitudes and frequencies describe the periodic oscillations in the decomposition of heart rate signals. This is assessed by the area (or power spectral density) of each component. Absolute values are expressed in ms^2^. Frequency domain indices include: the very low frequency band (VLF, <0.003-0.04 Hz, ms2); low-frequency power (LF, 0.04–0.15 Hz, ms^2^), a mixture of sympathetic and parasympathetic activity; high-frequency power (HF, 0.15-0.4 Hz, ms^2^), describing parasympathetic tone; and the low-high frequency power ratio (LF/HF), which reflects the balance of sympathetic and parasympathetic innervations. The LF/HF ratio has been shown to be a good indicator of the sympathovagal balance of the autonomic nervous system [26]. HF and LF power were calculated using fast Fourier transformation (FFT). FFT is a rapid and simple nonparametric method, characterized by specified frequency windows for the frequency components. Because absolute values are more sensitive than normalized indices in the analysis of 24 h HRV indices, absolute values of the direct measures were utilized and expressed in ms^2^ for the purpose of this study.

### Statistical analyses

Key characteristics and clinical parameters were expressed as Means (± SEM), with independent samples t-tests used to determine differences between the depression and control groups. Correlations between depression severity and the HRV indices, specifically SDNN and LF/HF, were assessed using Pearson’s analysis. SDNN reflects the total variability over the period of recording, hence has been shown to be the most significant prognostic value among time domain parameters [27]. For each participant, the presence of an arrhythmia was defined as the occurrence of single or paired atrial and/or ventricular premature beats as detected on the ECG. Significant between group differences in the prevalence of arrhythmias were calculated using the Chi-square test. SPSS19.0 software was used and the level of significance was set at *P*<.05.

## Results

### Demographic features

The key characteristics of the sample are displayed in Table 1. No statistically significant differences in demographic variables (age, sex, BP, smoking) were observed between the control and depression groups. Those in the depression group were slightly younger (40.9 years), had a higher mean systolic blood pressure (BP) and lower diastolic BP, and a higher rate of smoking than in the control group. Selective serotonin-reuptake inhibitors (SSRIs) were routinely used to treat to patients with major depression (dosing paroxetine 40 mg/d in 15 cases, escitalopram 20 mg/d in 18 cases, sertraline 100 mg/d in 20 cases).

**Table 1:** Demographic data between the control and depression groups (Mean ± SEM, n = 53) unless indicated

**Items**	**Control**	**Depression**
Ages (years)	42.1 ± 11.61	40.9 ± 10.5
(Ranges)	20–63	20–65
Gender (male/female) (n)	27/26 (51%/49%)	25/28 (47%/53%)
Resting SBP (mm Hg)	125 ± 20	130 ± 22
Resting DBP (mm Hg)	74 ± 9	70 ± 10
Smokers/non-smokers (n)	24/29 (45%/55%)	28/25 (53%/47%)

No significant differences in the demographic variables (age, sex, blood pressure, smoking) between the control and depression groups, were observed.

### The time domain indices of HRV

The comparison between the time domain indices of HRV in the two groups is illustrated in Table 2. In the depression group, the values of SDNN, SDANN, RMSSD, and PNN50 were significantly lower than that of the control group (*P*<.05).

**Table 2:** Comparison of time domain indices of HRV (Mean ± SEM, n=53)

**Indexes (ms^2^)**	**Depression**	**Control**
SDNN	93.13 ± 16.12**	110.57 ± 20.10
SDANN	84.34 ± 15.46*	91.09 ± 12.69
RMSSD	38.83 ± 8.82*	43.64 ± 11.01
PNN50	11.05 ± 7.32*	14.07 ± 6.72

Note: compared to control group, *P<.05,** *P*<.01.

### The frequency domain indices of HRV

The comparison between the mean frequency domain indices of HRV for the two groups is illustrated in Table 3. The values of LF and LF/HF were significantly higher in the depression group than the control group (*P*<.05), and the value of HF was significantly lower in the depression than the control group (*P*<.05).

**Table 3:** **Comparison of frequency domain indices of HRV (*Mean ± SEM, n=53*)**

**Indexes (ms^2^)**	**Depression**	**Control**
LF	19.17 ± 6.48*	16.41 ± 5.15
HF	18.64 ± 4.34*	20.95 ± 5.86
LF/HF	1.05 ± 0.35*	0.86 ± 0.41

Note: compared to control group, *P<.05.

### The relationship between depression severity and HRV indices

**Figure 1:**
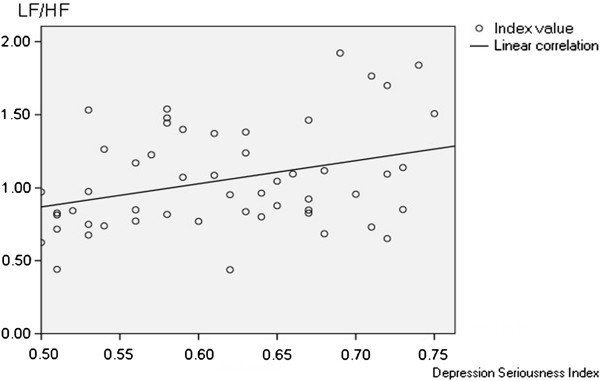
**The correlation between Depressive severity index and LF/HF.** Pearson relativity analysis shows a positive correlation between depressive severity index and LF/HF (*P*<.05, r = .335).

**Figure 2:**
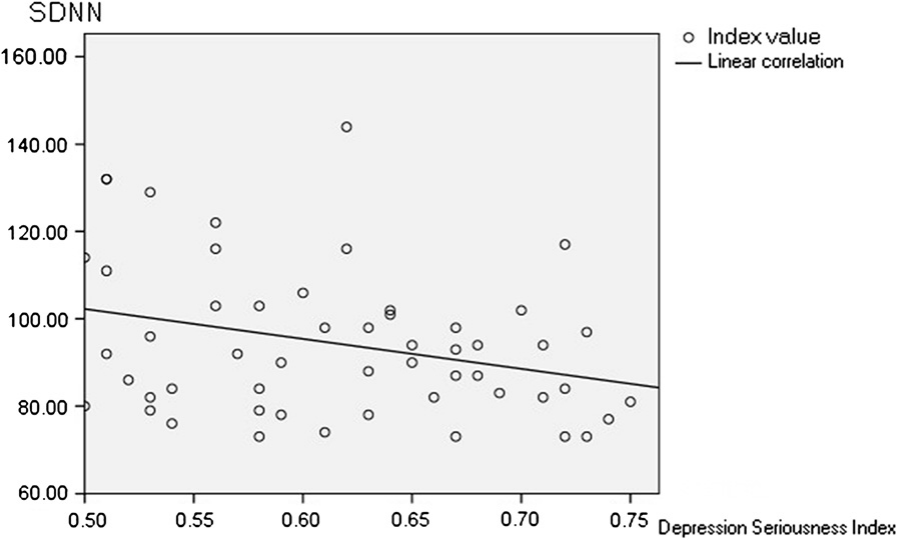
**The correlation between Depressive severity index and SDNN.** Pearson relativity analysis shows a negative correlation between depressive severity index and SDNN (*P*<.05; r = −.298).

Associations between the SDS depression severity index and HRV related indices are illustrated in Figures 1 &2. A linear relationship was evident between the depression severity index and the value of LF/HF (Figure 1). Pearson’s relativity analysis showed a positive correlation between the two groups (*P*<.05, r = .335). A negative correlation was observed between the depression severity index and the value of SDNN (Figure 2; *P*<.05; r = −.298), suggesting more severe depression was accompanied by a higher mean level of cardiac autonomic dysfunction.

### The prevalence of arrhythmia in depression

**Figure 3:**
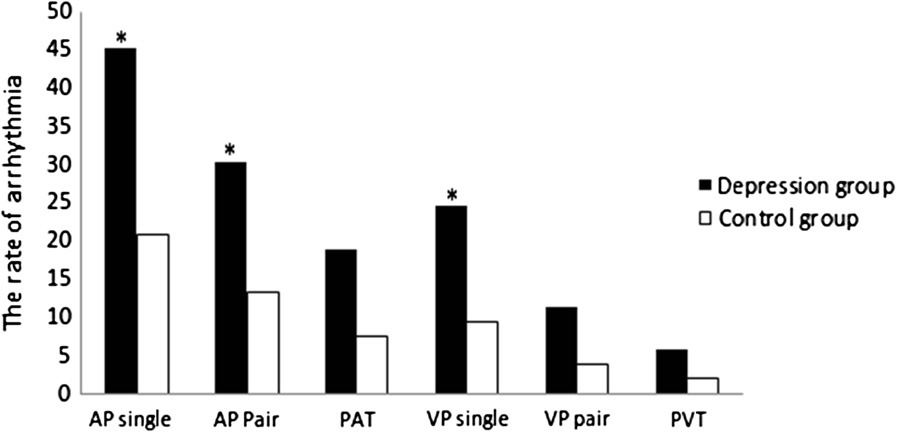
**Comparison the rates of arrhythmia between the depression and control group.** Note: Chi-square test, compared to control group, *P<.05.

There was a higher prevalence of arrhythmias, including single atrial premature beats, paired atrial premature beats and paired ventricular premature beats in the depressed group compared to the control group, *P* < .05. (Figure 3).

## Discussion

This study demonstrated that, when compared with a healthy control group, patients hospitalized for clinical depression exhibit altered cardiac nervous function, evidenced by a lower high frequency domain index and a higher low frequency domain index and LF/HF ratio. Increases in the severity of depressive symptoms were shown to exacerbate these trends. Arrhythmias, particularly supraventricular arrhythmias, were over-represented in depressed patients when compared to those without. The findings from this study are consistent with the hypothesis that cardiac autonomic dysfunction is experienced by individuals with depression, especially decreased parasympathetic nerve activity (as evidenced by decreased values of the time domain indexes). This is supported by other studies of patients with depression that have applied other assessment techniques. For example, Bi et.al [28] found that patients with depression experienced autonomic nervous dysfunction using sympathetic skin response measures.

There may be a number of explanations for this finding. A mood disorder may trigger a series of adverse cardiovascular factors that evoke pathophysiological changes, which have been detailed previously [29]. These study [2-4] have demonstrated a significant relationship between depression and cardiovascular risk factors that are known to lead to adverse outcomes. Battacharyya et al. [30] hypothesized that the relationship between depression and cardiovascular risk may be explained by enhanced parasympathetic control. We suggest that the decreased HF and increased LF and LF/HF ratio observed in our study (using frequency domain analysis) is indicative of reduced parasympathetic nerve activities and the imbalance of sympathetic and parasympathetic innervation, which may reflect dysregulation of sympathetic and parasympathetic coordination in depression. LF reflects the influence of sympathetic and parasympathetic nervous activity on heart rate fluctuation, and has relevance to vessel baroreflex effects. As such, enhanced vascular baroreceptor reflex resulting from emotional stress can induce increased LF. This is consistent with clinical manifestations in patients with depression who experience excessive tension, palpitation, chest tightness and other physical symptoms. HF may represent both vagus nerve activity and respiratory activity. However, Berger [31] found that while vagus activity was reduced in their group of depressed patients, there was no relationship between parasympathetic nervous activity and the frequency and rhythm of respiration. As there is a direct effect on HRV [32], where HRV is smaller if parasympathetic activity is lower and vice versa, we propose that parasympathetic nervous activity predominantly influences cardioregulatory function. Pathophysiological links between depression and cardiovascular system dysfunction include reduced HRV, changed sympathetic nerve activity, arrhythmia, and altered ventricular electrophysiological properties [15]. In examining HRV in depressed patients in our study, we speculated that the relative increase in sympathetic tone and corresponding reduction of parasympathetic tone may be a mechanism associated with this phenomenon and arrhythmia induction.

Our finding that a linear relationship exists between depression severity and HRV is consistent with previous research. Stein [33] found that cardiac patients with severe depression experienced significantly reduced HRV and increased mortality; a linear relationship between depression severity and level of cardiac autonomic dysfunction was observed. Agatisa [34] reported that recurrent episodes of elevated depressive symptoms were related to a greater risk of coronary and aortic atherosclerosis among those not yet diagnosed as having coronary disease. Some reports suggest the cardiac autonomic dysfunction in depressed patients may increase the risk of arrhythmia; which itself is a risk factor for coronary and other cardiovascular disorders. Indeed, we demonstrated a higher prevalence of arrhythmia in depressed patients compared with controls; a finding that may go some way to partially identifying a pathway by which depressed individuals are at increased susceptibility to CVD onset. That people with depression are more vulnerable to arrhythmias, especially supraventricular arrhythmias, such as single atrial premature beats, paired atrial premature beats and single ventricular premature beats, is in general agreement with the concept that there is a relationship between arrhythmia and emotional turbulence [35], which may be related to increased sympathetic nervous system activity [36]. Adrenergic secretion can increase the auto-rhythmicity of heart purkinje fibers, reducing the excited domain of the diastolic period and inducing sympathetic excitability. As a result, it may increase the probability of ventricular arrhythmia. Hyper-responsivity of sympathetic nervous activity may also constitute a risk factor for the development or progression of CVD [36].

Some of the inconsistencies in the evidence base surrounding the relationship between HRV and depression may be explained by variance in study design, assessment techniques and/or markers of autonomic functions used across studies [37]. In this study, we analyzed HRV and evaluated autonomic function through analysis of the time-domain indexes SDNN, SDANN, RMSSD and PNN50. The domain parameters of HRV used in this study are considered a reliable indicator for risk of malignant ventricular arrhythmias and sudden cardiac death when compared with ventricular late potentials, left ventricular ejection fraction, QT dispersion and the level of cardiac function [38,39]. Altered HRV may therefore be useful for predicting arrhythmia risk in patients with depression.

We acknowledge several limitations of this study. Ours was a sample taken from patients undergoing hospitalization and subsequent treatment for major depression. Based on the pre-determined selection criteria, they had few anxiety symptoms, a low risk of suicide, and an absence of acute myocardial infarction. While these criteria were employed in order to reduce the impact of confounding variables on our outcome of interest, this selection bias may affect the generalizability of our results. Secondly, the sample size was relatively small, limiting our ability to analyse the relationship between depression severity and HRV and perform meaningful sub-group analyses. Although it should be acknowledged that the markers of autonomic function are indirect measures, the comprehensiveness of the data including gold standard measures of depression using diagnostic psychiatric interviewing and HRV using domain parameters are strengths of this study. Thirdly, the cross sectional nature of the study does not allow examination of autonomic dysfunction across the disease course to determine whether remission affects this marker. Lastly, multiple comparisons were not undertaken as part of our statistical analyses; for example the effects of medication usage remain undetermined as all patients in the depressed group were receiving medication. We do acknowledge that SSRI have been shown to impact on cardiac rhythm, thus it is possible that using a sample comprising those on antidepressants may have diluted or alternatively, magnified our findings, compared with a sample from the general population, or using depressed individuals who were not on medication. Therefore, results should be interpreted with caution.

## Conclusions

Our findings suggest that depression is accompanied by dysfunction of the cardiac autonomic nervous system, and further, that depression severity is linked to severity of this dysfunction. Individuals with depression appear to be susceptible to premature atrial and/or ventricular disease.

## Abbreviations

CAD: Coronary artery disease; HAMD: Hamilton rating scale for depression; SDS: Self-rating depression scale; CMS: Chronic mild stress; HRV: Heart rate variability; SSRIs: Selective serotonin-reuptake inhibitors; HAMA: Hamilton anxiety scale; SDS: Self-rating depression scale; SDNN: Standard deviation of the NN intervals; SDANN: Standard deviation of 5 min averaged NN intervals; RMSSD: Root mean square of the successive differences; pNN50: Percentage of NN intervals > 50 ms different from preceding interval; VLF: Very low frequency band; LF: Low-frequency power; HF: High-frequency power; LF/HF: Ratio of low-high frequency power; FFT: Fast fourier transformation.

## Competing interests

Michael Berk has received Grant/Research Support from the NIH, Cooperative Research Centre, Simons Autism Foundation, Cancer Council of Victoria, Stanley Medical Research Foundation, MBF, NHMRC, Beyond Blue, Geelong Medical Research Foundation, Bristol Myers Squibb, Eli Lilly, Glaxo SmithKline, Organon, Novartis, Mayne Pharma and Servier, has been a speaker for Astra Zeneca, Bristol Myers Squibb, Eli Lilly, Glaxo SmithKline, Janssen Cilag, Lundbeck, Meat and Livestock Board, Merck, Pfizer, Sanofi Synthelabo, Servier, Solvay and Wyeth, and served as a consultant to Astra Zeneca, Bristol Myers Squibb, Eli Lilly, Glaxo SmithKline, Janssen Cilag, Lundbeck and Servier. The other authors declare that they have no conflicts interests.

## Authors’ contributions

YW and XL participated in the design of the study and performed the statistical analysis, MB, AO and AT participated in coordination and drafting of the manuscript. XZ carried out the studies tests. All authors participated in data interpretation, drafting of the manuscript and have read and approved the final manuscript.

## Pre-publication history

The pre-publication history for this paper can be accessed here:

http://www.biomedcentral.com/1471-244X/13/187/prepub
